# Research advances in pathogenic mechanisms and host response of mycoplasma pneumoniae pneumonia in children: a metabolomics perspective

**DOI:** 10.3389/fmed.2026.1763133

**Published:** 2026-02-11

**Authors:** Yuanhao Dai, Yujing Zhang, Xuran Guo, Lei Liang, Yage Zhao, Yongbin Yan

**Affiliations:** 1 First Affiliated Hospital of Henan University of Traditional Chinese Medicine, Zhengzhou, China; 2 Henan University of Chinese Medicine, Zhengzhou, China

**Keywords:** children, metabolomics, mycoplasma pneumoniae (M. pneumoniae), mycoplasma pneumoniae pneumonia, research advances

## Abstract

Mycoplasma pneumoniae pneumonia (MPP) is a common disorder that invades predominantly the school-aged children and adolescents globally. Given its nonspecific clinical manifestations at the initial stage and the significance of early identification of severe cases for clinical management, it highlights the necessity of diagnostic confirmation through laboratory testing. Recent advances in metabolomics have demonstrated significant potential in elucidating the pathogenic mechanisms of MPP. It enables an analysis of metabolic alterations in biological samples, thus providing a comprehensive understanding of disease-associated perturbations in metabolic networks, and offering novel insights into its etiology. Simultaneously, metabolomics can facilitate the discovery of potential biomarkers, thereby serving as valuable tools for early diagnosis and disease progression evaluation.

## 1 Introduction

Mycoplasma pneumoniae pneumonia (MPP), with the primary manifestation of tracheobronchitis, exhibits endemic and epidemic occurrence worldwide. During outbreaks, Mycoplasma pneumoniae (MP) accounts for up to 20 to 40% of community-acquired pneumonia in the general population, especially highly prevalent among school-aged children and adolescents (1). Young children with MPP often present with more complex clinical manifestations and prognoses, such as progression to refractory MPP or severe MPP (SMPP), accompanied by higher risk of co-infections in this age group (2) (3) (4). It underscores the utmost importance of timely and accurate diagnosis. However, the clinical diagnosis and treatment of pediatric MPP still face multiple bottlenecks, particularly in terms of deficiencies in early accurate classification and prediction of disease progression. Currently, the traditional detection methods commonly used in clinical practice are difficult to meet the aforementioned needs: although inflammatory markers (CRP and PCT) are convenient for detection, they have low specificity; imaging examinations (chest X-ray and CT) mainly focus on pulmonary morphological changes, exhibit insufficient sensitivity to minor lesions in the early stage of the disease, and are difficult to quantitatively evaluate treatment efficacy. Meanwhile, there is a lack of specific markers for early diagnosis, coupled with low sensitivity of specialized MP culture (5) (6), while MP-IgM antibody testing is prone to false positives due to the interference of human factors or autoantibodies (7). Moreover, immune chromatographic assays may induce missed diagnosis of infections in children with low antibody levels (8). In addition, nucleic acid amplification, biosensors, and next-generation sequencing are limited by technical challenges and difficulties in pediatric sample collection, even with advantages of convenience and sensitivity (7, 9). Consequently, in order to optimize diagnostic and therapeutic strategies, it is urgent to reveal the molecular mechanisms underlying disease progression.

Metabolomics has emerged as an alternative tool for studying biological mechanisms, which can facilitate a comprehensive understanding of cellular metabolic processes. This technique enables the elucidation of small and medium-sized metabolites in biological samples (e.g., fluids, cells, tissues), thus revealing correlations between metabolic profiles and disease pathology, offering insights into disease progression and host responses (10). Both untargeted and targeted metabolomics can achieve the identification of disease-associated metabolites and novel biomarkers, given their employment of platforms such as liquid chromatography-mass spectrometry (LC-MS), gas chromatography-mass spectrometry, and nuclear magnetic resonance (NMR). This methodology holds promise for predicting disease progression and optimizing clinical management. For instance, metabolomics has been applied in newborn genetic metabolic disease screening (11), with the identification of metabolites linked to asthma susceptibility in infants with bronchiolitis (12), and detection of biomarkers for evaluating *Helicobacter pylori* eradication efficacy in children (13).

Pathogen detection or inflammatory factor expression remain the major concerns in prior research, without comprehensive presentation of the host metabolic-immune crosstalk. In contrast, the pathogenesis of MPP involves host metabolic remodeling induced by MP infection, including lipid metabolism disorders and amino acid metabolism imbalance. Metabolomics can provide crucial evidence for deciphering the “pathogen-host” interaction as this tool enables a direpct capture of these dynamic changes. For MPP, metabolomics can not only achieve early accurate identification and classification of the disease by analyzing characteristic metabolic profiles, but also in-depth reveal the mechanism of metabolic disorders in the body caused by MP infection. Meanwhile, it can use metabolic markers as quantitative indicators to evaluate treatment efficacy and prognostic risks, thereby providing a new molecular targeting perspective for the precision diagnosis and treatment of pediatric MPP. These endow metabolomics with irreplaceable and unique value in pediatric MPP research. Furthermore, there is still an insufficient development of pediatric metabolic systems, and the impact of MPP on their metabolic networks may exhibit age-specific characteristics. By employing metabolomics, it is possible to accurately capture such development-related metabolic differences, holding significant value for understanding the pathogenesis and progression of MPP. Accordingly, the present review summarizes current findings on metabolic alterations in MPP and explores potential biomarkers to elucidate its pathogenesis and progression.

## 2 Current research status of metabolomics in MPP

Research on MPP has revealed key changes in the processing of fats and proteins by the human body. In an investigation comparing blood samples from healthy children, children with other infections, and those with MPP, children with MPP revealed distinct patterns in molecules related to fat metabolism, such as glycerophospholipids, triglycerides (TGs), and sphingolipids (14–16). Among these, small fatty acids (e.g., acetate and isobutyrate) may contribute to the diagnosis of MP infection and prediction of disease severity (16). Differences in fat-related molecules between mild and severe MPP cases may also be related to the presence of complications outside the lungs, which may offer evidence to clarify the worsening of the disease (14). Protein-building blocks (amino acids) also play a critical role in MPP. Children with MPP show changes in 13 amino acids in their blood, which may effect energy production, immune function, tissue repair, etc. (15). For example, molecules such as L-hydroxyproline and serine rise alongside increased white blood cell counts, possibly damaging lung cells (15). Urine tests in MPP patients identified 73 altered molecules, most tied to amino acid pathways. Meanwhile, elevated threonine levels stood out as a potential warning sign for MPP (17). In the comparison of mild and severe MPP cases, shifts in amino acids (e.g., L-serine and L-cysteine) in urine highlighted disruptions in sugar metabolism, vitamin production, and antioxidant systems (18). Key molecules such as glutamate and glycine were proposed to be reliable to distinguish MP-infected patients from healthy individuals (19). Altogether, these findings emphasize the role of fat-amino acid imbalance in driving MPP progression, which may provide additional evidence for better diagnosis and therapies.

## 3 Lipid metabolism and MPP

### 3.1 Glycerophospholipids

Glycerophospholipids are key components of cell membranes involved in cellular signaling, including phosphatidylcholine (PC) and phosphatidylethanolamine (PE), the most abundant phospholipids in mammalian cells. These molecules have been identified to undergo significant alterations in many diseases. Pathogens involving MP can exploit host-derived lipid membranes to evade the immune system during early infection, thereby disrupting host cell membrane function and structure through extracellular infection (15). Specifically, MP can scavenge essential lipids via the membrane-anchored protein P116, hence modulating its membrane composition to adapt to the host. This mechanism enables glycerophospholipids on the MP cell membrane to share certain antigenic components with host cells, thereby allowing MP to evade the host immune surveillance. Simultaneously, MP can synthesize key lipids, including phosphatidylcholine and sphingomyelin, to meet its basic metabolic requirements (20). Meanwhile, phospholipids and their derived metabolites (e.g., glycerol) serve as the main source of carbon and energy for MP to colonize lung epithelial cells. During this process, hydrogen peroxide, generated by glycerol-3-phosphate oxidation, is a major virulence factor contributing to MP pathogenesis (21). It is fully consistent with the “glycerophospholipid dysregulation” signature identified in pediatric metabolomic studies on MPP. Compared to healthy children, patients with MPP had elevated levels of PC, PE, lysophosphatidic acid (LPA), and lysophosphatidylcholine (LysoPC), suggesting MP infection acting as a de-stabilizer of the lipid bilayer of host cell membranes (14). It is likely that MP, after invading alveolar epithelial cells, may proliferate extensively by utilizing phospholipids as the primary substrate, thereby leading to increased consumption of phospholipids and the presence of “glycerophospholipid dysregulation” in pediatric MPP. Moreover, under the catalysis of phospholipases, phospholipids are extensively converted into key inflammatory mediators (e.g., lysophospholipids, prostaglandins, arachidonic acid, etc.). Beyond amplifying the local inflammatory response. It may also drive the pathogenesis and progression of MPP (22). Furthermore, glycerophospholipids can mediate immune responses in inflammatory diseases. Their metabolism relies on hydrolysis by phospholipases to maintain balance. Reduced phospholipase activity can promote the accumulation of metabolic byproducts. In studies on SMPP, treatment reduced PC levels in mouse lungs, increasing PE. This shift promoted the release of anti-inflammatory unsaturated fatty acids (e.g., arachidonic acid), as well as enhanced phospholipase synthesis and hydrolysis, reducing phospholipid buildup (23). Lysophosphatidylethanolamine (LysoPE) can generate LysoPC, PE, and PC, all of which are critical components of the pulmonary surfactant lipid bilayer. LysoPE can modulate neutrophil activity and phospholipase A2 release to trigger inflammation (24). LysoPC possesses pro-inflammatory and cytotoxic properties. Elevated intracellular LysoPC levels correlate with apoptosis induction, endoplasmic reticulum and mitochondrial stress (25), as well as immune cell activation (e.g., neutrophils, and macrophages), which may accelerate pro-inflammatory cytokine and chemokine release (26). Specifically, LysoPC 18:0 may worsen the airway damage through the exacerbation of inflammation by stimulating reactive oxygen species production (27). Abnormal plasma phospholipid levels thus indicates the severity of lung inflammation in MP infection (15), aligning with the established understanding of immune activation-involved MP pathogenesis (28).

### 3.2 Sphingolipids

Sphingolipid metabolism can induce the release of bioactive molecules such as ceramides, sphingosine, sphingosine-1-phosphate, and ceramide-1-phosphate, functioning to regulate cell proliferation, differentiation, apoptosis, and inflammation (29). MP infection may disrupt sphingolipid metabolism in human lung cells (e.g., A549), altering ceramide and sphingomyelin production, depending on bacterial load and infection duration. At specific pathogen concentrations, MP may mediate key enzymes [(e.g., serine palmitoyltransferase and acid sphingomyelinase (ASM)], further influencing sphingolipid dynamics (30). Ceramides, central to sphingolipid signaling, can activate apoptosis pathways post-infection, threby balancing immune responses and preventing excessive inflammation (31). During cellular stress, ceramides replace cholesterol in membranes, forming tightly packed microdomains to induce the aggregation of signaling receptors (e.g., CD95, and DR5), amplifying apoptosis signals via the ASM activity (32, 33). Failure to form these ceramide-rich platforms may lead to uncontrolled release of inflammatory cytokines to exacerbate tissue damage. In children with MPP, there existed significant difference in the plasma levels of ceramide Cer (d18:0/18:0) when compared with healthy controls (HC), suggesting a role in disease pathology, despite the lack of confirmation of the existence of these membrane structures in MPP (14). Elevated ceramides can also induce oxidative stress and apoptosis in lung cells by accumulating superoxides, while C2-ceramide and other synthetic analogs can enhance prostaglandin E2 production via cyclooxygenase upregulation, linking sphingolipids to cytokine-driven inflammation (29, 34, 35). Additionally, sphingosine, another sphingolipid derivative, exhibits antimicrobial activity against bacteria and viruses in mucosal barriers, with its role in MP infection remained to be unveiled (36). Collectively, sphingolipids are critical players in MPP pathogenesis, which can mediate immune regulation, apoptosis, and inflammation, with implications for therapeutic targeting.

### 3.3 TTGs

TGs are crucial energy storage molecules that are hydrolyzed by TG lipases to produce diacylglycerol, further converting into phosphatidylinositol (PI) via phosphatase activity. Following mono-, di-, or tri-phosphorylation by lipid kinases, PI may generate seven phosphorylated derivatives that regulate membrane trafficking, protein recruitment, cytoskeletal organization, endocytosis, and autophagy (37). Prior research has documented distinct alterations in specific plasma lipid species in MPP patients, including TG (18:3/20:4/20:4), TG (14:0/18:2/22:6), and PI variants such as PI (16:0/18:1), PI (16:0/18:2), PI (18:0/22:6), and PI (18:0/22:5). It can be interpreted that MP infection may disrupt the host energy metabolism and lipid processing (15). MP can also induce lipid droplet formation in Raw264.7 macrophages, potentially hijacking host lipids for its own growth, while increasing the risk of metabolic disorders (e.g., hypertriglyceridemia)in the host (38).

## 4 Amino acid metabolism and MPP

**Figure 1:**
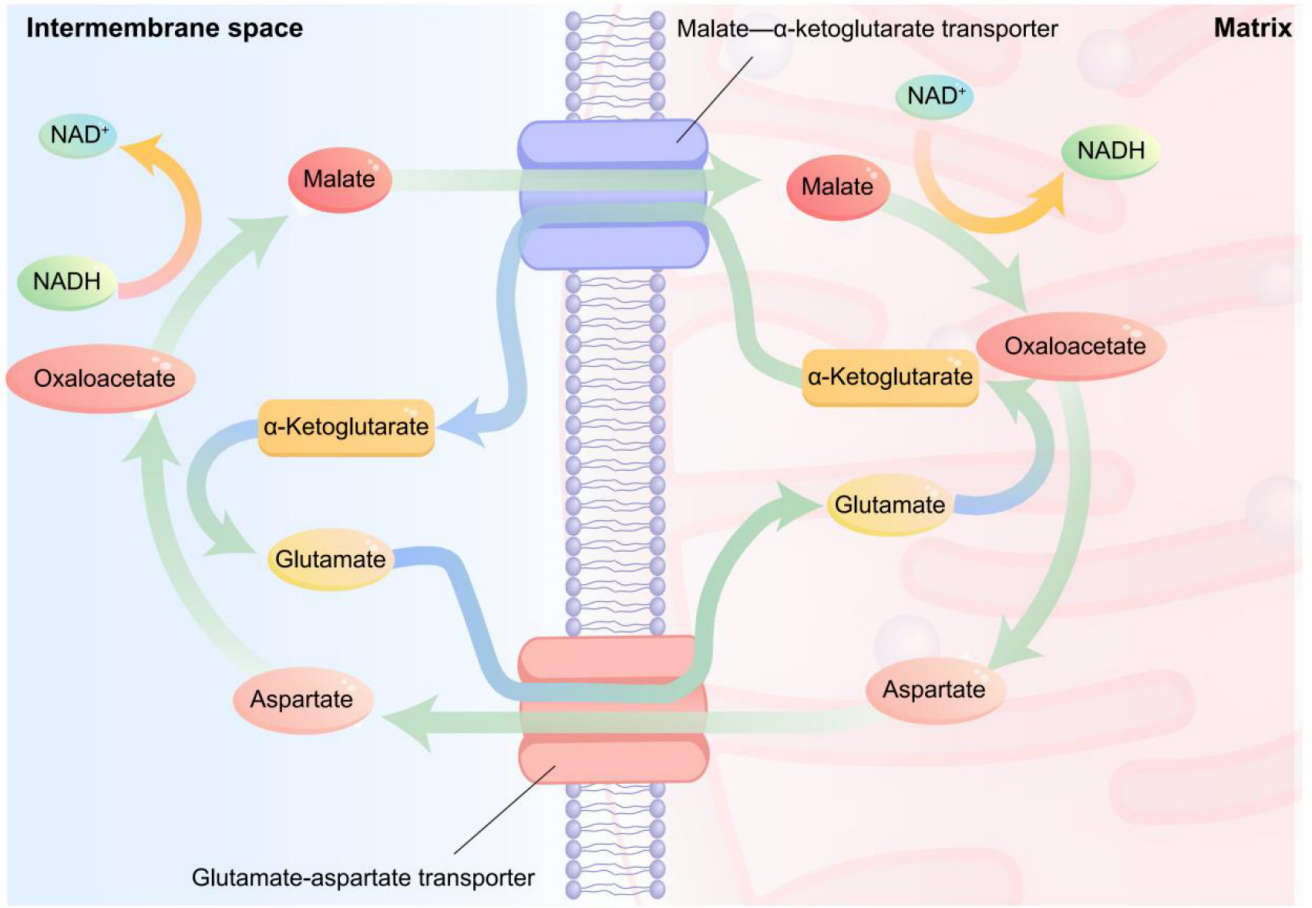
The malate-aspartate shuttle When NADH generated by cytoplasmic glycolysis cannot directly penetrate the inner mitochondrial membrane, it is reduced to malate from oxaloacetate by cytoplasmic malate dehydrogenase. Malate then enters the mitochondrial matrix via the malate–α-ketoglutarate carrier and is subsequently oxidized to NADH and oxaloacetate by mitochondrial malate dehydrogenase. Oxaloacetate is converted to aspartate by aspartate transaminase, which is transported back to the cytoplasm via the aspartate–glutamate carrier to regenerate oxaloacetate.

As critical components of the human immune system, amino acids are essential for both normal bodily functioning and disease processes (39). Changes in amino acid metabolism may suggest the immune response of the human body to infections, tissue damage, and repair efforts. For example, Wu et al. (40) identified a pathway involving glycine, serine, and threonine in regulating carbohydrate metabolism and energy use to combat oxidative stress. Critically, dysregulated amino acid metabolism also has a relationship with the occurrence of lung diseases such as chronic obstructive pulmonary disease, where bacterial infections may worsen lung function and reduce amino acid (e.g., asparagine, citrulline, glutamine, histidine, methionine, serine, and threonine) levels in the blood (41). As evidenced previously, significant disruptions in amino acid metabolism have been confirmed in MP infection (15, 18, 19). Key pathways include the production of valine, leucine, and isoleucine, which are altered in asthma and asthma with MP co-infection. The malate-aspartate shuttle, a cellular energy pathway, has been proposed as a distinct feature of MP infection (42) (Figure 1), potentially tied to its role in oxidative stress, cell death, and immune regulation, all of which may disturb the function of lung cell barriers (39).

### 4.1 Glycine

Following, MP infection, reduced glycine levels may impair neutrophil-mediated inflammatory responses and contribute to epithelial dysfunction (15). Glycine has been recognized to be an essential metabolite for maintaining lung extracellular matrix integrity, exerting pivotal role in combating oxidative stress (43). Ma et al. (44) demonstrated that dietary glycine supplementation in mice reduced lipopolysaccharide (LPS)-induced lung inflammation, and its mechanism was related to the decreased neutrophil and macrophage infiltration, collagen deposition, inflammatory cytokine release, and alveolar cell apoptosis. Similarly, through the mediation of tight junction protein levels and cellular localization, glycine supplementation in piglets alleviated oxidative stress-induced epithelial barrier dysfunction and apoptosis (43). Altogether, glycine supplementation may mitigate infection-induced alveolar damage, offering new therapeutic strategies for MPP.

### 4.2 Serine

MP infection can elevate plasma serine levels in children (18, 19). Serine serves as a precursor in multiple biosynthetic pathways, including nucleotide synthesis, S-adenosylmethionine production, NADPH generation (via the *de novo* serine synthesis pathway), and glutathione synthesis (45). Infection has been proven to increases intracellular serine levels, thereby promoting S-adenosylmethionine-mediated reactions and enhancing pro-inflammatory cytokine secretion in macrophages (18). Conversely, serine deprivation would reduce LPS-induced IL-1β release and glutathione synthesis in murine peritoneal macrophages. Moreover, the suppression of *de novo* serine synthesis could lower LPS-driven pro-inflammatory cytokine levels and improve the survival in sepsis models (46). Consequently, in SMPP, inflammatory cytokine release may accelerate serine metabolism, creating a feedback loop amplifying the severity of inflammation (18).

## 5 Potential biomarkers for MPP

Comparative plasma analyses between MPP patients, HC, and children infected with other pathogens (IDC) identified three metabolites (568.5661, 459.3493, and 411.3208) with high diagnostic value. Specifically, Notably, metabolite 411.3208 (4a-formyl-5a-cholest-8, 24-dien-3b-ol) could effectively discriminate MPP from both HC and IDC in the validation set, with sensitivity and specificity both approaching 100%. It might be a highly specific diagnostic biomarker for MPP, offering pivotal evidence for differentiating MPP from other infectious diseases in pediatric practice clinically. Metabolite 459.3493 demonstrated sensitivities of 85.7% (vs. HC) and 71.4% (vs. IDC), with specificities of 87.5% and 89%, respectively. Meanwhile, metabolite 568.5661 [Cer (d18:0/18:0)] showed sensitivities of 93.8% (vs. HC) and 89.3% (vs. IDC), with specificities of 96.4% and 90% (14). In another analysis of carboxyl- and carbonyl-containing metabolites in serum using 5-Br-2-HP derivatization-LC-MS, there were significant changes in MP-infected children, including upregulated 6-keto-PGF1α (AUC = 0.92) and 15(S)-HEPE (AUC = 0.89), while downregulated 12(S)-HHTrE (AUC = 0.99) and NA-Trp (AUC = 0.95). These metabolites show promise as biomarkers for MP infection. In urine samples, MPP patients were measured with lower acetyl phosphate levels compared to HC, potentially linked to disruptions in taurine and hypotaurine metabolism. Conversely, the observed elevated 2, 5-dioxovaleric acid levels possibly led to arginine and proline metabolic dysregulation. Such urinary biomarkers, detectable through simple sampling, hold significant promise for early MPP diagnosis (17). However, current studies on these potential biomarkers are still in the exploratory stage. Notably, their clinical applications are hindered owing to two critical limitations, i.e., relatively small sample sizes and lack independent external validation with a multicenter, large-sample design. There is currently an incomplete elucidation of the specific biological functions of some biomarkers (e.g., metabolite 411.3208) and their definite roles in MPP/Hence, further in-depth investigation is necessitated to uncover their origins, metabolic pathways, and correlations with clinical outcomes (i.e., clinical symptoms, disease severity, treatment response, and prognosis).

Hui et al. (22) provided evidence for phospholipids as potential predictors of MP mixed infection, given that phospholipids exhibited the lowest level in MP-mixed virus group (*P* < 0.05), with RSV coinfection being the most common subtype. Significantly, it aligned with our previous observation of “glycerophospholipid dysregulation” in MPP, as DPPG (a key phospholipid) may inhibit viral infection by interfering with receptor-ligand binding. Furthermore, viral infection-induced inflammatory cell activation can promote PLA2-mediated phosphatidylglycerol hydrolysis to LPC—an effector that impairs alveolar surfactant activity—ultimately leading to reduced glycerophospholipid levels. Collectively, in addition to being a signature of MP mixed infection, phospholipid metabolism disorders may also serve as a mechanistic link between MP-virus coinfection and lung dysfunction.

By integrating proteomic and metabolomic approaches, Wang et al. (47) identified seven inflammation-related KEGG pathways. The specific pathways were arginine and proline metabolism, Fanconi anemia pathway, histidine metabolism, taurine and hypotaurine metabolism, phenylalanine metabolism, p53 signaling pathway, and tyrosine metabolism. The involved proteins mainly included CKMT1A, MAOA, ERCC1, MLH1, APAF1, MAOB, ALDH3A1, SERPINB5, FMO5, and SFN. Pearson correlation analysis was performed between these proteins and differential metabolites from metabolomic pathways [i.e., L-Arginine, palmitic acid, guanine, 4-nitrophenol, and 8-(3-Octyl-2-oxiranyl) octanoic acid]. Consequently, 8-(3-Octyl-2-oxiranyl)octanoic acid exhibited positive correlations with CKMT1A, FMO5, SERPINB5, ALDH3A1, SFN, MAOA, and MLH1. Palmitic acid was negatively correlated with GGT3P and APAF1, but positively correlated with SFN, MAOA, and MLH1. L-Arginine was negatively correlated with APAF1, while positively correlated with SERPINB5, CKB, and MLH1. Guanine exhibited a strong positive correlation with FMO3, CKMT1A, FMO5, SERPINB5, ALDH3A1, SFN, MAOB, ERCC1, and MAOA. Therefore, integrative analysis of multi-omics data may provide a more comprehensive perspective for potential MPP biomarker screening. In the future, there is still a need to identify a panel of MPP biomarkers with higher specificity and sensitivity through large-scale cohort studies and in-depth integration of multi-omics data.

## 6 Technical variability of metabolomics platforms and discussion

Given different technical principles of each platform, there exists a certain degree of variability among different metabolomics platforms. Gas chromatography-mass spectrometry (GC-MS) boasts core advantages in detecting volatile, semi-volatile, and thermally stable metabolites, including short-chain fatty acids, fatty acid methyl esters, carbohydrate derivatives, and amino acid derivatives. However, complex pretreatment involving derivatization is required in terms of non-volatile and thermally unstable metabolites (e.g., most glycerophospholipids, sphingolipids, and macromolecular peptides). Variations in derivatizing reagent dosage, reaction temperature, and reaction time can trigger deviations in the quantitative results of the same metabolite (up to 2–5 times) and even render some metabolites undetectable (48). Significantly, NMR enables unbiased detection of hydrophilic small molecules with a molecular weight < 1,000 Da (e.g., glucose, lactic acid, amino acids, and organic acids), allowing the quantification of dozens of core metabolites simultaneously. Compared to LC-MS and GC-MS, this technology features simple sample pretreatment without no complex enrichment needed, a high degree of process standardization, and remarkably lower pretreatment-induced variability. Nevertheless, NMR has low sensitivity, imposing a challenging when detecting low-abundance metabolites (e.g., certain lipids and inflammatory mediators), and is sensitive to high-concentration matrices (e.g., serum proteins), which may induce signal overlap (49). LC-MS, on the other hand, excels in detecting non-volatile, medium-to-high polarity, and thermally unstable metabolites. It is particularly adept at analyzing low-abundance hydrophobic metabolites, in addition to detecting metabolites with a wide polarity range (from hydrophilic to hydrophobic), including lipids, alkaloids, peptides, and carbohydrates. But its range of detection depends on the chromatographic column and ionization method, which may yield certain detection bias that prevents simultaneous coverage of all metabolite classes. Besides, chromatographic separation efficiency and matrix effects are two major causes related to a compromised quantitative accuracy, leading to poor reproducibility between different laboratories (50).

In summary, due to the complexity of metabolites, a single platform is not suitable to detect all metabolite types, highlighting the presence of “platform-specific” biomarkers. The integration of different analytical tools can yield comprehensive metabolomic results. In studies on biomarker screening for MPP, pediatric MPP samples are characterized by significant trace amounts (e.g., fingerstick blood, and sputum samples) as well as diverse and complex matrices (e.g., serum contains high-abundance proteins, sputum contains mucins, and urine contains metabolic wastes). Different sample types vary in their suitability for metabolomics platforms, requiring targeted selection. Specifically, NMR should be prioritized to identify common metabolic biomarkers across sample types during preliminary screening of various pediatric MPP samples (serum, sputum, urine, and fingerstick blood). LC-MS or GC-MS should be further used for targeted and accurate quantification based on different biomarkers. A synergistic application of these three platforms can maximize the coverage of metabolic characteristics of pediatric MPP and enhance the clinical translation value of the screened biomarkers.

Currently, most metabolomic studies on pediatric MPP exhibit an obscure defect of lacking multi-platform comparison data, given their adoption a single platform (predominantly LC-MS) typically (51), which may challenge the clinical validation of biomarkers. Future studies should carry out methodological comparison to clarify the advantages and limitations of different platforms in metabolomic analysis of pediatric MPP. Moreover, a unified metabolite annotation database should be constructed to reduce annotation difference-induced result inconsistencies. A standardization of sample pretreatment processes is also required in the future, and multi-platform-integrated cross-validation should be relied upon to lower detection bias of a single platform. Additionally, there may be significant platform-specific differences in the normal reference ranges of the same biomarker, making it difficult to form a unified “pediatric MPP metabolic characteristic profile”.

## 7 Research limitations

It should be noticed that there are still several limitations in current metabolomic studies on pediatric MPP. First, the generally small sample size was a prominent issue in current research. In the majority of single-center, small-sample exploratory studies, thet statistical power of the research results might be weakened, which may restrict an accurate identification of the real changes in the pediatric MPP metabolic profile. It may also produce negative impact on the reliability and universality of the screened potential biomarkers. The second limitation might be the heterogeneity of research subjects. Existing studies focused primarily on adults or animal studies, necessitating further consideration of the specific differences in the immune system, lung development, and metabolic system development of children. After MP infection, the peak secretion level and duration of inflammatory factors in children differ from those in adults. As important metabolic regulatory signals, inflammatory factors can directly affect the response direction of lipid metabolism and amino acid metabolism, thus leading to significant differences in the metabolic disorder profile between children and adults after infection; From the perspective of pulmonary function and tissue metabolic characteristics, the lung tissue of children is in a stage of rapid development, with active metabolism of pulmonary parenchymal cells. The pulmonary parenchymal inflammation induced by MP infection can more directly disrupt the energy metabolism (e.g., mitochondrial dysfunction) and substance synthesis metabolism (e.g., abnormal synthesis of pulmonary surfactant) of lung tissue; The metabolic network of the pediatric body is not yet fully established and has strong plasticity, making it more prone to compensatory metabolic changes under the stress of Mycoplasma pneumoniae infection. Moreover, the metabolomic results would be interfered by several factors, such as large age range of MPP children, varied degrees of disease severity (e.g., common type vs. severe type), the presence of mixed infections, and differences in therapeutic intervention measures. At present, some studies failed to fully consider these variables or conduct detailed stratified analyses, which may mask the characteristic metabolic changes in specific subgroups of children. Furthermore, there was still a poor and insufficient interpretation of metabolomic data and elaboration of mechanisms. Major direction of research was the discovery of differential metabolites, with relatively few discussions on the precise participation of these metabolites in the pathogenesis of MPP mechanistically, their upstream and downstream regulatory networks, and the specific molecular mechanisms between them and the host immune response. There is a lack of in-depth functional verification experiments to reveal the causal relationships of metabolic disorders with the occurrence and development of diseases. Differences also existed among different studies in sample collection and processing, metabolomic detection platform selection, data preprocessing, and statistical analysis methods. It may impede an effective comparison and integration of results from different studies, which is not conducive to forming a unified understanding of MPP metabolic characteristics. Simultaneously, the lack of longitudinal dynamic studies is another shortcoming. Given the cross-sectional design, most studies were difficult to capture the dynamic evolution law of metabolic profiles at different stages of MPP (e.g., acute phase, and recovery phase) and could not clarify the associations of metabolite changes with disease progression, outcome, and prognosis. The particularity of the pediatric population also brought certain challenges to relevant research. For example, due to ethical restrictions, it was difficult to obtain samples, especially samples from healthy control children. With regard to the above, larger-sample, multi-center, prospective cohort studies are required in the future, coupled with multi-omics integration analysis and functional experiments. It may eventually facilitate a more comprehensive and in-depth elucidation of the metabolic mechanisms of MPP, and promote the application of metabolomics in the clinical diagnosis and treatment of MPP.

## 8 Summary

Mtabolomics has currently significantly advanced our understanding of pediatric MPP. Existing comparative studies between MPP patients and HC or pathogen-infected controls have identified key metabolites and pathways linked to disease development, particularly involving disruptions in lipid metabolism (e.g., glycerophospholipids, and sphingolipids) and amino acid metabolism (e.g., serine, and glycine). Beyond revealing broad impact of MP infection on metabolic networks, these findings also highlight the role of amino acids and lipids in assessing disease severity. Metabolomics has further deepened our insights into MP pathogenesis, revealing promise for early diagnosis and disease monitoring in the context of measuring specific metabolites in plasma or urine. Among these, lipid species (e.g., ceramides and lysophospholipids), along with amino acid profiles, have emerged as potential biomarkers. In the future, there is a need to further validate these biomarkers and promote corresponding translation into clinical practice. On these basis, we can acquire valuable data to facilitate the development of novel therapeutic strategies for MPP by employing targeted interventions in specific pathways, such as modulating serine metabolism to reduce inflammation or leveraging dysregulated lipids like ceramides.

In conclusion, metabolomics provides fresh perspectives on MPP mechanisms, laying the groundwork for innovative diagnostic and therapeutic approaches, and underscoring its potential for clinical translation.
